# Potential Cost-Effectiveness of Schistosomiasis Treatment for Reducing HIV Transmission in Africa – The Case of Zimbabwean Women

**DOI:** 10.1371/journal.pntd.0002346

**Published:** 2013-08-01

**Authors:** Martial L. Ndeffo Mbah, Eric M. Poolman, Katherine E. Atkins, Evan W. Orenstein, Lauren Ancel Meyers, Jeffrey P. Townsend, Alison P. Galvani

**Affiliations:** 1 School of Public Health, Yale University, New Haven, Connecticut, United States of America; 2 Department of Family Medicine, Oregon Health and Science University, Portland, Oregon, United States of America; 3 Emory University School of Medicine, Atlanta, Georgia, United States of America; 4 Section of Integrative Biology, The University of Texas at Austin, Austin, Texas, United States of America; 5 Department of Ecology and Evolutionary Biology, Yale University, New Haven, Connecticut, United States of America; Centre Suisse de Recherches Scientifiques, Cote D'ivoire

## Abstract

**Background:**

Epidemiological data from Zimbabwe suggests that genital infection with *Schistosoma haematobium* may increase the risk of HIV infection in young women. Therefore, the treatment of *Schistosoma haematobium* with praziquantel could be a potential strategy for reducing HIV infection. Here we assess the potential cost-effectiveness of praziquantel as a novel intervention strategy against HIV infection.

**Methods:**

We developed a mathematical model of female genital schistosomiasis (FGS) and HIV infections in Zimbabwe that we fitted to cross-sectional data of FGS and HIV prevalence of 1999. We validated our epidemic projections using antenatal clinic data on HIV prevalence. We simulated annual praziquantel administration to school-age children. We then used these model predictions to perform a cost-effectiveness analysis of annual administration of praziquantel as a potential measure to reduce the burden of HIV in sub-Saharan Africa.

**Findings:**

We showed that for a variation of efficacy between 30–70% of mass praziquantel administration for reducing the enhanced risk of HIV transmission per sexual act due to FGS, annual administration of praziquantel to school-age children in Zimbabwe could result in net savings of US$16–101 million compared with no mass treatment of schistosomiasis over a ten-year period. For a variation in efficacy between 30–70% of mass praziquantel administration for reducing the acquisition of FGS, annual administration of praziquantel to school-age children could result in net savings of US$36−92 million over a ten-year period.

**Conclusions:**

In addition to reducing schistosomiasis burden, mass praziquantel administration may be a highly cost-effective way of reducing HIV infections in sub-Saharan Africa. Program costs per case of HIV averted are similar to, and under some conditions much better than, other interventions that are currently implemented in Africa to reduce HIV transmission. As a cost-saving strategy, mass praziquantel administration should be prioritized over other less cost-effective public health interventions.

## Author Summary

Evidence from epidemiological and clinical studies supports the hypothesis that genital infection with S*chistosoma haematobium* increases the risk of becoming infected with HIV among women in sub-Saharan Africa. Praziquantel is an oral, nontoxic, inexpensive medication recommended for treatment of schistosomiasis, which might be able to prevent the development of genital schistosomiasis. We constructed a mathematical model of female genital schistosomiasis and HIV infections, which we calibrated using epidemiological data from Zimbabwe. We used this model to investigate the potential cost-effectiveness of mass drug administration with praziquantel as an intervention strategy for reducing HIV transmission in sub-Saharan Africa. We showed that mass drug administration with praziquantel may be a timely, innovative, and cost-saving intervention strategy for HIV prevention in sub-Saharan Africa. As a cost-saving strategy, mass drug administration with praziquantel should be prioritized over other less cost-effective public health interventions. Our findings indicate the possible benefit of scaling up schistosomiasis control efforts in sub-Saharan Africa, and especially in areas were S*chistosoma haematobium* and HIV are highly prevalent.

## Introduction

Sub-Saharan Africa continues to bear a disproportionate share of the global HIV burden [1]. Parallel to HIV, schistosomiasis is highly prevalent in sub-Saharan Africa, where approximately two-thirds of schistosomiasis cases result from urinary and genital tract infections caused by *Schistosoma haematobium* [2], [3]. Epidemiological studies have observed that female genital schistosomiasis (FGS) is associated with increased odds of having HIV [4]–[7]. Thus, mass preventive chemotherapy against schistosomiasis may not only reduce schistosomiasis morbidity and mortality in sub-Saharan Africa, but could simultaneously offer an innovative approach to HIV/AIDS prevention [8], [9].

There are three lines of evidence that indicate to an association between FGS and an elevated risk of HIV infection. Firstly, there is a strong statistical association between FGS and HIV transmission. Several cross-sectional epidemiological studies have reported that in sub-Saharan Africa, the region most heavily affected by the HIV/AIDS pandemic, women with FGS have a three- to four-fold increased odds of having HIV compared to women without FGS [5], [7]. Secondly, it is physiologically plausible that FGS elevates susceptibility to HIV infection through its lesions and chronic inflammation of genital tract, as well as chronic immunomodulatory effects [4], [10]. Thirdly, the presence of schistosomal lesions is common in the vulva and the lower vagina of FGS infected women before puberty [11], [12], making it likely that the schistosomal infection typically precedes HIV infection. Thus, collectively, the strong statistical association between FGS and HIV, the biologic plausibility of the association, and the temporal association suggest that FGS infection exacerbates HIV transmission.

Praziquantel is a highly effective anti-schistosomal chemotherapy agent against schistosomal morbidity that may be able to prevent FGS and the clinical manifestations associated with enhanced HIV susceptibility [12]–[14]. We conducted a cost-effectiveness analysis of mass praziquantel administration for HIV/AIDS prevention from the perspective of health payers, such as national government or international donors, which are the major providers of both mass schistosomiasis treatment and HIV antiretroviral therapy in sub-Saharan Africa [8], [15], [16]. For this, we constructed a model of the joint dynamics of HIV and FGS among sexually active individuals that we parameterized with epidemiological data from a cross-sectional study of rural Zimbabwean women [3], [7]. We calculated cost-effectiveness ratios of a potential large-scale intervention based on praziquantel as a preventive anthelminthic chemotherapy in terms of reducing HIV incidence. We found that mass preventive chemotherapy of schistosomiasis might prove not only very cost-effective, but even cost-saving in preventing HIV infection in *S. haematobium*-endemic areas.

## Methods

### Overview

To estimate the potential cost-effectiveness of preventing HIV infection in sub-Sahara Africa through mass treatment of schistosomiasis, we constructed a mathematical model for genital schistosomiasis and HIV infections in the adult population (aged 15–49, corresponding to the standard age-range for WHO reporting of HIV prevalence [17]). We parameterized the model by applying a Bayesian inference approach to cross-sectional epidemiological data on FGS and HIV among rural Zimbabwean women [3], [7]. We calculated the cost-effectiveness from a health care system perspective, because the Zimbabwean health care system and international donors are the primary providers of treatment costs for HIV [15], [18]–[20]. Only direct medical costs to the health provider were considered, including the costs of mass administration of praziquantel and lifetime treatment costs of an HIV infection. We quantified the cost-effectiveness of praziquantel in terms of HIV cases averted and averted medical care costs over the duration of the intervention. Costs and benefits were discounted at an annual rate of 3%, according to WHO recommendations [21].

### Dynamic Model of HIV-FGS Infection

**Figure 1:**
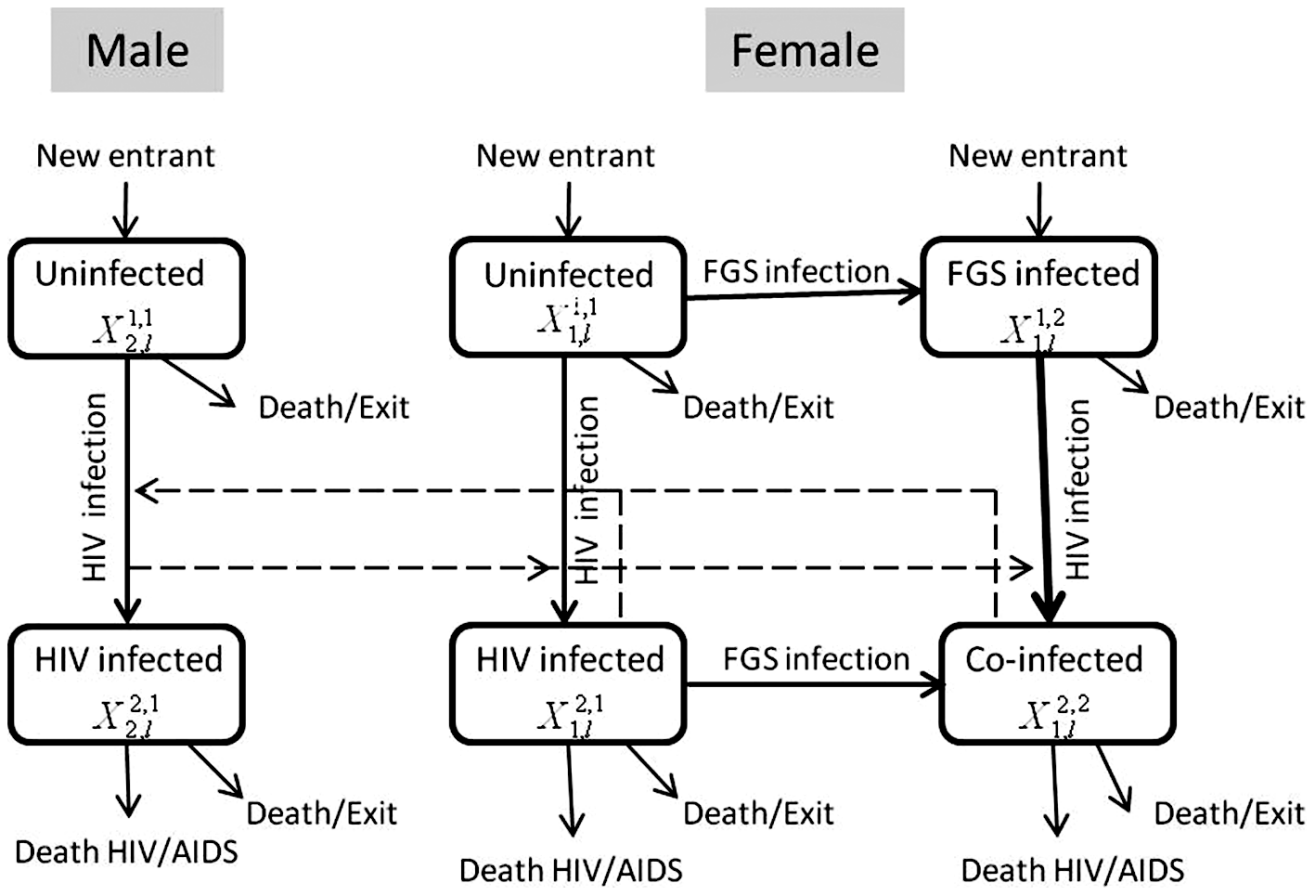
Outline of FGS-HIV transmission model. The flow between epidemiological classes for the transmission dynamics of FGS and HIV. Individuals enter the system at age 15, and exit at age 50. 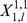, 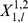, 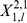, and 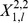 denote, respectively, the number of women uninfected by HIV and FGS, infected with FGS, infected with HIV, and infected with both HIV and FGS. 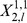
and 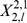 denote, respectively, the number of men uninfected and infected with HIV.

Initially, we modeled a Zimbabwean population with no praziquantel treatment. We divided the population into males and females, and high and low sexual activity risk groups defined according to rate of sexual partner change. The state variable of the model are given by 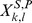: 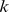 is gender (1 = female, 2 = male), 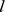 is the sexual activity group (1 = high risk, 2 = low risk), 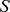
is the HIV infection status (1 = susceptible, 2 = infected), and 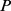 is the FGS infection status for women (1 = non-infected, 2 = infected). For men 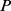 was always equal to 1. Individuals enter the model at the onset of sexual activity (assumed to be age 15), with a proportion of women infected with FGS. From age 15 to 49, women acquire FGS and/or HIV at rates dependent on their infection status of each disease. As re-infection rates of schistosomiasis are high in *S. haematobium*-endemic areas [12], [22], we assumed no natural recovery from FGS. The acquisition of HIV was modeled as a function of the rate of partner change, the mixing between individuals of the different sexual risk groups, the number of sex acts in partnerships per year, and the rate of HIV transmission per sex act (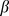). The rate of forming sexual partnerships is allowed to decline with HIV mortality to model potential behavior change as a response to the HIV epidemic [23]. FGS infection is assumed to elevate the risk of HIV transmission per sex act by a factor 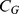, which was parameterized from epidemiological data on HIV-FGS co-infection. Therefore, the rate of HIV transmission from men to FGS-infected women is 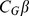. We assumed that FGS is primarily acquired in childhood [13], [22]. To determine *S. haematobium* prevalence, we developed a model for *S. haematobium* dynamics (see Appendix for details) from which we quantify FGS prevalence in the HIV model. The compartmental diagram in Figure 1 illustrates the flow of individuals as they face the possibility of acquiring each infection.

We used Bayesian Markov Chain Monte Carlo (MCMC) [24] to fit the HIV-FGS model to epidemiological data on FGS and HIV prevalence and co-infection among rural Zimbabwean women [3], [7]. The MCMC approach allowed us to estimate uncertain epidemiological parameters by combining prior information about these parameters from epidemiological studies (Appendix), empirical HIV-schistosomiasis data, and dynamic model prevalence predictions. We determined the likelihood function of our MCMC approach from empirical data [3], [7], assuming normal distributions for HIV and FGS prevalence and lognormal distribution for the odds ratio. Using this approach, we derived a posterior distribution for each epidemiological parameter (Table 1) for which the model gives the best-estimated trajectory for HIV and FGS prevalence and odds ratio of the association between HIV and FGS. Our model predicted the annual prevalence of HIV/AIDS in a Zimbabwean setting over a baseline period of 10 years starting in 2000. To validate the model, we compared these predictions of HIV prevalence to observed HIV prevalence from Zimbabwean antenatal clinics [17].

**Table 1:** Estimates of the parameters used in our dynamic HIV-FGS model (Figure 1).

Variable	Meaning (units)	Prior distribution [ref]	Posterior distribution: median (95% CI)
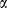	Annual growth rate rural population	0.034 [29]	N/A
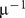	Duration of sexual activity (year^-1^)	1/35	N/A
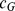	Enhanced HIV transmission due to FGS	Uniform(0,20)	5.9 (3.8–9.1)
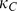	Probability of FGS given childhood infection	Uniform(0.33,0.75) [8]	0.47 (0.38–0.56)
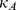	Probability of acquiring FGS during adulthood	Uniform(0.005,0.05)	0.008 (0.006–0.009)
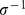	Duration of HIV infection (years)	Uniform(7.5,12.5) [33, 48]	10.7 (8.1–12.1)
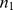	Number of sex acts in partnerships per year for high-risk group	Uniform(15,150) [48]	128 (95–148)
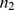	Number of sex acts in partnerships per year for low-risk group	Uniform(50,248) [48]	69 (28–137)
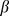	HIV transmission rate per sex act	Uniform(0.0006,0.004) [23], [39]	0.0022 (0.0009–0.003)
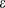	Mixing between sexual risk groups	Uniform(0.2,0.9) [49]	0.44 (0.22–0.62)
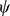	Extent to which males determine the pattern of sexual partnerships formation	Uniform(0.2,0.8) [48]	0.67 (0.50–0.78)
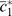	Initial partner change rate: women (year^-1^)	Triangular(0.66,2.4,0.9) [48]	1.27 (0.7–2.2)
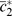	Initial partner change rate: men (year^-1^)	Triangular(1.1,3,1.2) [48]	1.9 (1.2–2.8)
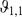	Fraction of women in high-risk group	Uniform(0.05,0.6) [48]	0.20 (0.15–0.24)
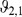	Fraction of men in high-risk group	Uniform(0.10,0.75) [48]	0.34 (0.22–0.49)
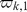	Relative rate of partner change: high-risk versus low-risk group	Uniform(1,100) [48]	10.7 (2.5–21.8)
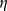	Reduction rate of partner change	Uniform(1,50)	6.5 (3.5–9.0)
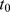	Year HIV epidemic starts	Uniform(1978,1985) [48,50]	1981 (1980–1982)

These parameter estimates produced the best fit of our dynamic model to epidemiological data for HIV and FGS prevalence and co-infection among rural Zimbabwean women [3], [7]. The dynamic model was fit to these data using a Markov Chain Monte Carlo method, which allowed us to calculate distributions of possible values for each of these parameters. We present here the mean of these distributions and their associated 95% credible intervals. The Brooks-Gelman-Rubin (BGR) method was used to monitor convergence of iterative simulations. Convergence was achieved when the upper limit of the credible interval of the BGR diagnostic statistic for a given parameter <1.2 [51].

### Praziquantel Intervention

Praziquantel is a highly effective anti-schistosomal therapy agent against schistosomal morbidity [8], with no serious or long-lasting side effects [25], [26]. The WHO recommends that mass administration of praziquantel be undertaken annually in schistosomiasis-endemic areas, targeting school-age children [27]. We investigated a strategy for praziquantel administration, where praziquantel is annually administered to school-age children (ages 5–14 years) (school-age strategy). We assumed that all praziquantel-treated girls reaching age 15 uninfected with FGS were less likely to develop FGS [14], [22] than those who had been infected in childhood. We considered two scenarios for the potential effect of mass praziquantel administration for reducing HIV incidence either in terms of reducing the risk of HIV transmission or reducing FGS prevalence. In the first scenario, we assumed that women who have received praziquantel treatment during childhood have a reduced risk of HIV transmission (30–70%) relative to FGS infected women who did not receive treatment during childhood, thus reducing 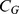 by 30–70%. In the second scenario, we assumed that women who have received praziquantel treatment during childhood have a reduced FGS prevalence relative to those who did not receive treatment (30–70%). We simulated each mode of action to predict the potential effect of mass treatment of schistosomiasis on reducing HIV incidence at the population level. In these analyses we assumed that the Zimbabwean population aged 15–49 years old was 4 million in 2000 [28], [29].

### Costs

To estimate the funding required to implement the alternative strategies of praziquantel treatment, we used the WHO dose for praziquantel (2.5 tablets of 600 mg per child per year) [27] which costs US$0.08 [30], while the total delivery cost per child treated, including delivery, training, social mobilization, capital equipment, and administrative costs, was US$0.21 (US$0.06–2.23) [31], [32]. We used US$0.29 (0.008+0.21) as a base value for the total cost of treatment per individual. Medical costs for HIV treatment and care included provider-initiated testing (diagnostic and routine offer of testing), treatment and prophylaxis for opportunistic infections, antiretroviral therapy, laboratory monitoring of antiretroviral therapy, and palliative care. The lifetime treatment costs of an HIV infection were based on recent estimates of the costs in sub-Saharan Africa of US$3469 in 2004US$ at a 5% discount rate [33]. For a 3% discount rate, this cost becomes US$3695. The antiretroviral therapy coverage in Zimbabwe was assumed to be 34% (28–40%) [34]. The Zimbabwean government expenditure on health, other than HIV related spending, was estimated to be US$26 (US$12–US$41) per capita annually [16], [35]. To compute the non-HIV/AIDS health expenditure per HIV case averted, we assumed that average age of HIV/AIDS acquisition among Zimbabwean is 25 years old [36], and the life expectancy at age 25 is 28 years [37]. All costs were discounted at 3% and given in US$2004 [21].

### Cost-Effectiveness Framework

We calculated the potential cost-effectiveness of mass praziquantel administration to school-age children for reducing HIV transmission. The status quo was to be no mass treatment of schistosomiasis, as is currently the case in Zimbabwe. We measured the effectiveness of the intervention in terms of the number of HIV cases averted during a baseline intervention period of 10 years. We measured cost-effectiveness in terms of program costs per HIV case averted and averted medical care costs over the ten-year intervention period as the base line duration, which was also varied to assess the impact of the duration of intervention on its cost-effectiveness.

### Sensitivity Analysis

To identify the contribution of each model parameter to the variability of the number of HIV cases averted, we calculated the partial rank correlation coefficients (PRCCs) [38]. PRCC quantifies the degree of monotonicity between a specific input parameter and an outcome measure. For this purpose, we used a Latin Hypercube Sampling [38] to sample 10,000 estimates of input parameters from the posterior distributions of the epidemiological parameters (Table 1) and distributions of the cost and efficacy of mass administration of praziquantel as well as antiretroviral therapy coverage and non-HIV/AIDS health expenditure (Table S1).

## Results

### Dynamic Model

**Figure 2:**
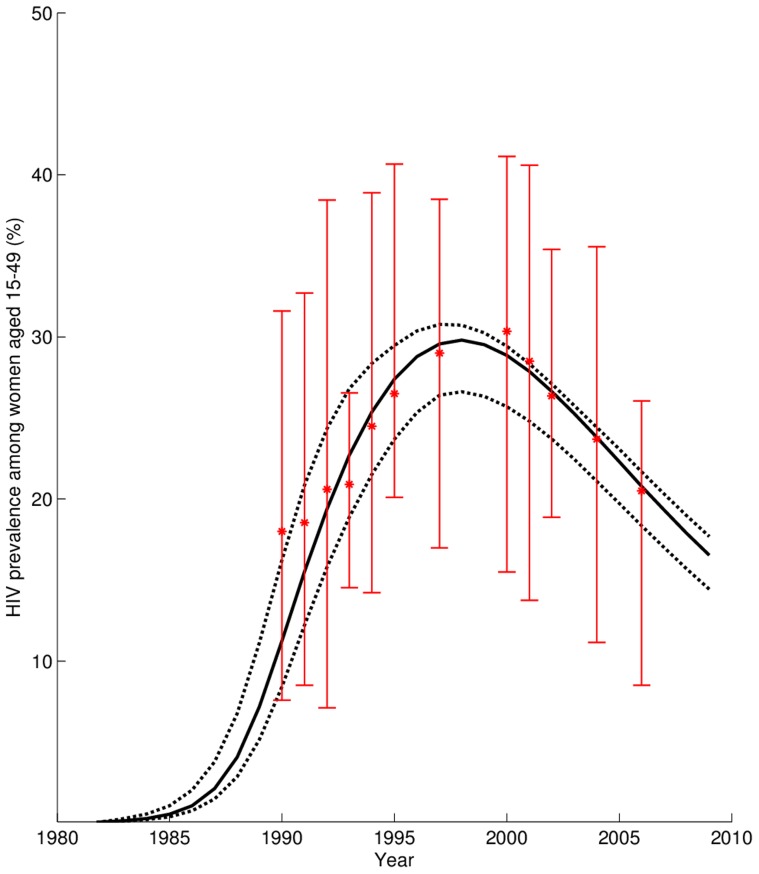
Comparison of model predictions to Zimbabwe antenatal clinic data for non-urban areas [17]. The solid line represents the yearly HIV prevalence as estimated by our model from baseline epidemiological parameters (dotted lines are the 2.5^th^ and 97.5^th^ percentile values). Empirical HIV prevalence is shown as stars (error bars are the 95% confidence intervals). The model was validated from antenatal clinic data not originally used for model parameterization.

To fit our dynamic model to the epidemiological HIV/FGS data from rural Zimbabwe [3], [7], we used a Markov Chain Monte Carlo (MCMC) method to draw values of epidemiological parameters from prior distributions based on estimates available in the literature. We derived a posterior distribution for each epidemiological parameter (Table 1) for which the model gives the best-estimated trajectory for HIV and FGS prevalence (Table 2). We estimated the value for 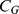, the coefficient by which FGS increases the risk of HIV transmission per sexual act, as 5.9 (95% CI: 3.8–9.1) The lower bound of the 95% credible interval (CI), for the estimate of 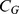, lies above one, consistent with the hypothesis that FGS enhances the risk of HIV transmission. Moreover, these estimates are in agreement with those of epidemiological and clinical studies that have observed that the presence or history of genital ulcers is associated with greater susceptibility to HIV transmission per sex act of 1.4 to 19.5 relative to the absence of genital ulcerative disease [39]. We validated the projected HIV prevalence of our model against the Zimbabwean antenatal clinic data from 1990–2006 (Figure 2), which had not been used to fit the model.

**Table 2:** Model fit to data.

	Kjetland et al [3], [7]	Model
**FGS prevalence** (95% CI)	46.1% (41.8–50.5)	45.5% (43.6–48.1)
**HIV prevalence** (95% CI)	28.1% (24.0–32.5)	29.6% (26.0–32.0)
**Odds Ratio** (95% CI)	2.1 (1.2–3.5)	2.1 (1.5–2.8)

FGS prevalence, HIV prevalence, and odds ratio of the association between FGS and HIV from the 1999 cross-sectional epidemiological study among rural Zimbabwean women [3], [7] and model predictions.

### Cost-Effectiveness

**Figure 3:**
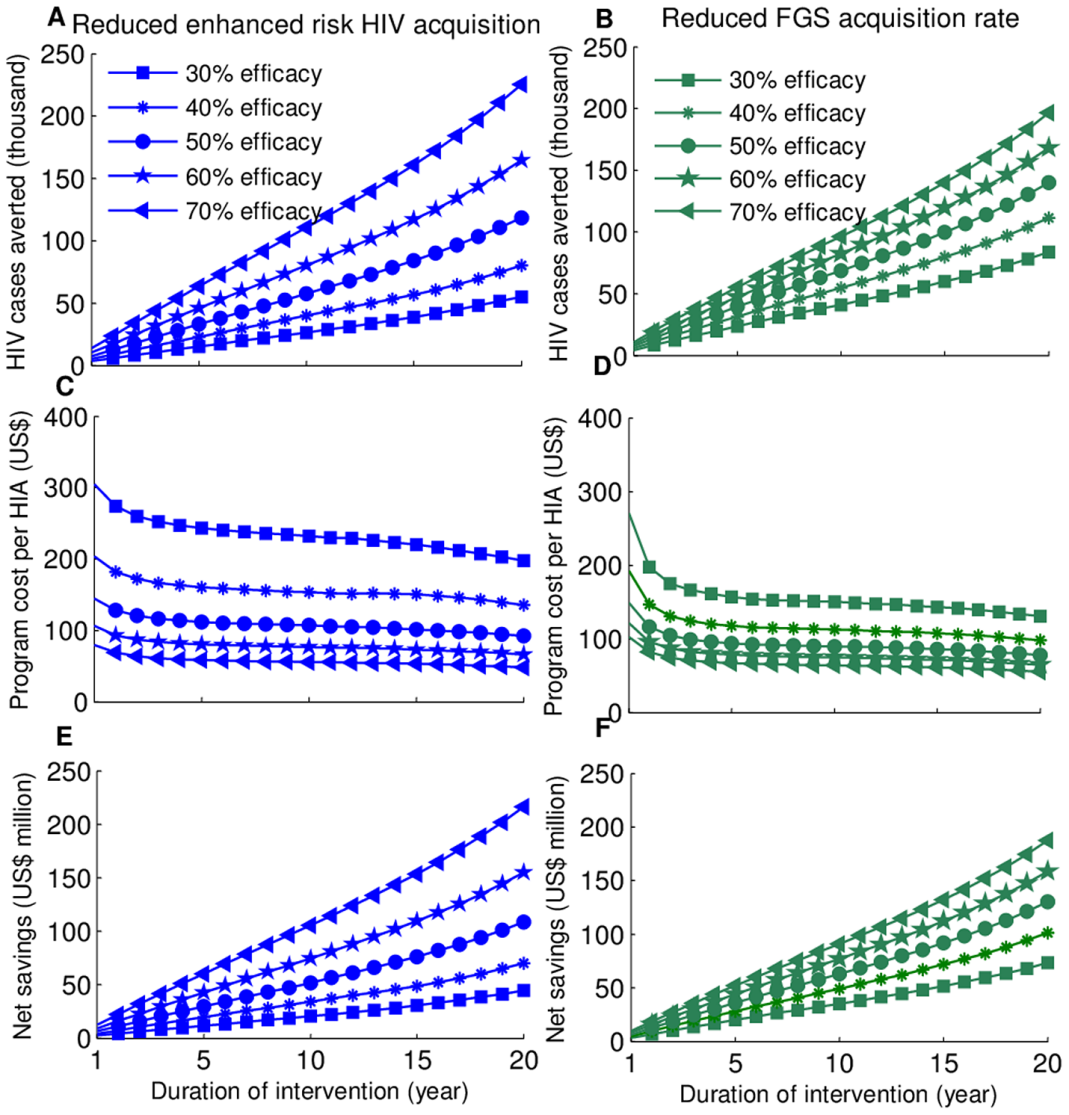
Cost-effectiveness of school-age intervention for the base case analysis. The number of HIV cases averted (A,B), the cost per HIV cases averted (C,D), and the net savings (E,F) were computed for different efficacies of mass praziquantel administration in reducing FGS (B,D,F) and the mitigated risk of HIV infection per sexual act (A,C,E).

We used the estimated mean values of the epidemiological parameters of the dynamic model input to calculate the incidence of HIV and FGS from 2000 to 2009. We considered two scenarios for the mechanism through which annual praziquantel administration for may reduce HIV transmission. In the first scenario, we assumed that for women who received praziquantel during their childhood, treatment reduces their enhanced risk of HIV transmission per sex act. In this scenario, the model predicted that, for an efficacy of 30%, annual administration of a single dose of praziquantel to all school-age children could avert 21,120 (95% CI: 11,000–55,395) cases of HIV at a program cost of US$259.31 per HIV case averted over a ten-year period (Figure 3). When adjusted for averted medical care costs, the net saving for school-age strategy compared to the status quo was estimated to be US$15.8 (95% CI: -US$13.0–50.7) million (Figure 3). For an efficacy of 70%, annual praziquantel administration to school-age children could avert 106,200 (95% CI: 74,025–207,230) cases of HIV at a program cost of US$51.68 per HIV case averted over a ten-year period (Figure 3). When adjusted for averted medical care costs, the net saving for school-age strategy compared to the status quo was estimated to be US$101.4 (95% CI: US$39.0–233.6) million (Figure 3). In the second scenario, we assumed that women who received praziquantel during their childhood have a reduced FGS prevalence. In this scenario, the model predicted that, for an efficacy of 30%, annual praziquantel administration to school-age children could avert 41,500 (95% CI: 34,000–79,190) cases of HIV at a program cost of US$131.94 per HIV case averted over a ten-year period (Figure 3). When adjusted for averted medical care costs, the net saving for school-age strategy compared to the status quo was estimated to be US$36.4 (95% CI: US$3.4–77.5) million (Figure 3). For an efficacy of 70%, annual praziquantel administration to school-age children could avert 96,945 (95% CI: 79,370–185,260) cases of HIV at a program cost of US56.50 per HIV case averted aver a ten-year period (Figure 3). When adjusted for averted medical care costs, the net saving for the school-age strategy compared to the status quo was estimated to be US$92.3 (95% CI: US$38.0–200.0) million (Figure 3).

### Sensitivity Analysis

**Figure 4:**
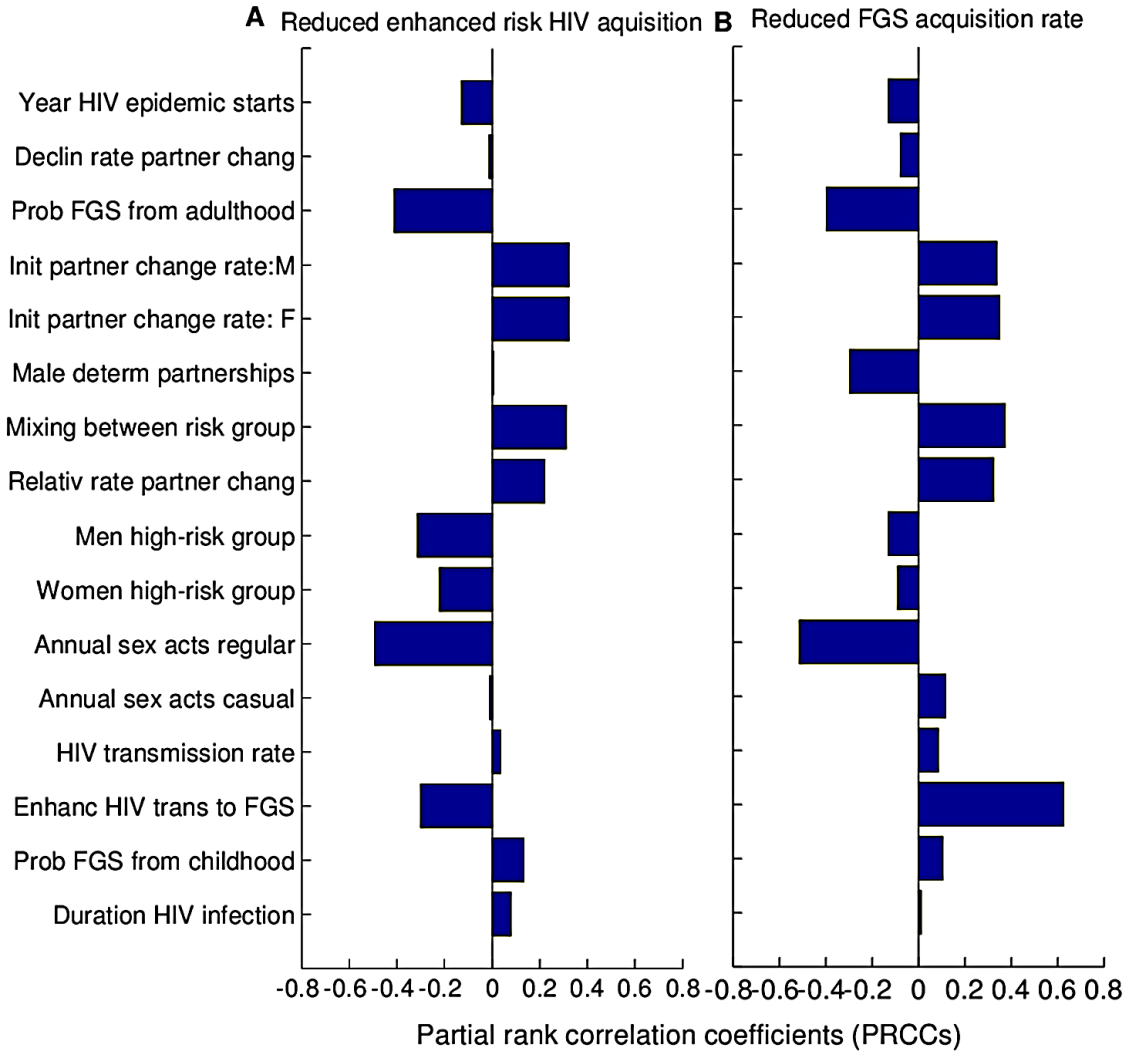
Partial rank correlation coefficients (PRCCs). A parameter was considered to be important in affecting the effectiveness of mass drug administration with praziquantel for impact on HIV transmission if |PRCC|>0.4. Specifically, probability of acquiring FGS from adult infection and the annual number of sex acts in low risk partnerships were the most important parameters for the first scenario (A), and the coefficient by which FGS enhances HIV transmission rate per sex act and the annual number of sex acts in low-risk partnerships were the most important parameters for the second scenario (B).

For the first scenario of the mechanistic basis of praziquantel in which the elevated risk of HIV acquisition is mitigated, variation in the number of HIV cases averted was primarily driven by 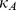, the probability of acquiring FGS from adult infection, and 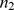, the annual number of sex acts in low risk partnerships (Figure 4). For the second scenario, when mass praziquantel reduces FGS prevalence, variation in the number of HIV cases averted was primarily driven by 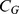, the coefficient by which FGS enhances HIV transmission rate per sex act, and 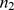, the annual number of sex acts in low-risk partnerships (Figure 4), highlighting the importance of this parameter to our analysis.

## Discussion

Our analysis demonstrated that mass administration of praziquantel could possibly be a cost-saving intervention for preventing HIV infection in Zimbabwe. For a wide range of efficacies, annual treatment of school-age children between 5 and 14 years could result in saving between US$15.8 and US$101.4 million medical costs over a ten-year period for a program cost of US$51.68–259.31 per HIV case averted. We found that even the indirect benefits of reducing HIV transmission alone may be sufficient to make mass praziquantel administration a cost-saving intervention. As a cost-saving intervention, mass praziquantel administration should be prioritized over other less cost-effectiveness public health interventions. Given the additional health benefits of reducing schistosomiasis morbidity that were not considered, because our model does not explicitly capture the complex age-structure dynamics of *S. haematobium* [40], [41], our cost-effectiveness results are conservative. Moreover, our model does not take into account the WHO recommendation of reducing the frequency of mass administration of praziquantel as schistosomiasis prevalence falls below 10% [27]. Accounting for this potential reduction in the frequency of mass treatment would reduce costs and might further enhance the cost-effectiveness of mass praziquantel administration. The results of our cost-effectiveness model may be applicable to other regions of sub-Saharan Africa with socio-cultural and epidemiological settings similar to those of Zimbabwe. The donation of praziquantel by pharmaceutical companies and international donors would substantially contribute to reducing the cost of mass schistosomiasis treatment in sub-Saharan Africa, making it even more cost-effective for HIV prevention.

Cost-savings arise at the interface of the low cost of praziquantel, its high efficacy, and elevated risk of HIV among FGS-infected women. Mass treatment of schistosomiasis also compares favorably to other biomedical interventions against HIV transmission. Treatment of sexually transmitted diseases is estimated to cost between $304 and $514 per HIV case averted [42]. Male circumcision is estimated to cost between $174 and $2808 per HIV case averted [43]. Antiretroviral therapy, while cost-effective in preventing HIV-associated morbidity and mortality, is less efficient in preventing new HIV infections (cost per HIV case averted>$20,000) [42].

Our analyses indicate the potential for mass treatment of schistosomiasis as an innovative and cost-saving public health tool for preventing HIV infections in sub-Saharan Africa. Given that epidemiological and clinical data on FGS dynamics are scarce, our model does not account for the potential natural acquisition of partial immunity to FGS, with age, among adult women [44]. Furthermore, the cost-effectiveness of mass administration of praziquantel on reducing HIV transmission in sub-Saharan Africa should also be explored in the context of increasing antiretroviral therapy coverage and other HIV prevention measure such as male circumcision. We anticipate that an increase in antiretroviral therapy coverage would result in increasing the initial annual national spending on HIV treatment [23], thus making mass treatment of schistosomiasis more cost-effective for HIV prevention. In the long term, the national spending on HIV treatment may decrease with HIV prevalence [23], thereby reducing the cost-effectiveness of mass treatment of schistosomiasis. Allying schistosomiasis control with HIV/AIDS control programs might offer synergetic opportunities for administration to reduce costs of delivery and increase the coverage of implementation.

Praziquantel prevents long-term schistosomiasis induced morbidity by killing egg-laying schistosomes in the host [27]. In schistosomiasis-endemic areas, individual treatment is of minimal benefit to the recipient as the risk of post-treatment reinfection remains very high [29], [45]. To significantly impact schistosomiasis morbidity in endemic areas, mass praziquantel administration should be administered periodically to entire communities or targeted to school-age children, which is the age-group at highest risk of infection [27]. However, intervention strategies that are based exclusively on mass praziquantel administration are likely to be unsustainable and to establish an indefinite chain of dependence on a pharmacological intervention. To complement the effectiveness of schistosomiasis control program and ensure sustainability of control efforts, mass praziquantel administration should be coupled with improvement of sanitation facilities, clean water supply, and health and hygiene education [27], [46].

Epidemiological studies have shown that women infected with genital schistosomiasis have a three- to four-fold increased odds of having HIV compared to women without genital schistosomiasis [5], [7]. However, each of these studies is cross-sectional and as such they can only determine an association, rather than a cause-effect relationship, between genital schistosomiasis and HIV infection. Definitive proof of a cause-effect relationship can only be established through longitudinal studies. A prospective randomized controlled study to assess the effect of praziquantel treatment on HIV incidence has been proposed as a necessary step toward developing a new protocol to treat schistosomiasis for HIV prevention [47]. There is pressing need for future epidemiological studies and control trials will fill the gaps in our knowledge on the association between schistosomiasis and HIV. Our analyses indicate that future control trials should not only aim to provide information on the relative risk of HIV acquisition given schistosomiasis infection and the efficacy of praziquantel treatment to reduce the increased susceptibility to HIV infection, but should also document the rates of FGS acquisition among children and adults as well as confounding sexual behavior, which we found to be fundamental in determining the effectiveness and cost-effectiveness of mass praziquantel administration. Genital schistosomiasis may interact with other risk factors, such as other genital ulcerative diseases and sexual behavior, exacerbating the risk of HIV infection.

Our results suggest that mass treatment of schistosomiasis in sub-Saharan Africa would not only have direct health benefits of reducing schistosomiasis infections, it may also avert cases of HIV and reduce the cost burden on the Zimbabwean medical system. By considering the impact of this neglected disease on HIV transmission, public health efforts can be expanded to include a broader set of cost-effective control strategies.

## Supporting Information

Protocol S1 **Mathematical details and model parameters.**
(DOC)
