# Supplementary material for: Potential Cost-Effectiveness of Schistosomiasis Treatment for Reducing HIV Transmission in Africa – The Case of Zimbabwean Women
Source: PLoS Negl Trop Dis. 2013 Aug 1;7(8):e2346. doi: 10.1371/journal.pntd.0002346 (PMC3731236; doi:10.1371/journal.pntd.0002346)
Supplement: Protocol S1 — Mathematical details and model parameters. (DOC) [file pntd.0002346.s001.doc]

**Web Appendix**

Potential cost-effectiveness of schistosomiasis treatment for reducing HIV transmission in Africa: The case of Zimbabwean women

Martial L Ndeffo Mbah, Eric Poolman, Katherine E Atkins, Evan W Orenstein, Lauren Ancel Meyers, Jeffrey P Townsend, and Alison P Galvani

**Supplementary information: Methods**

***Model Structure:***

We developed a mathematical model of the interplay between HIV and female genital schistosomiasis (FGS). We used data from a cross-sectional study in rural Zimbabwe^1,2^ to determine the posterior distributions of model parameters through a Bayesian analysis. Based on these parameter estimates, we quantified the potential cost-effectiveness of mass administration of praziquantel as an HIV preventive intervention.

***Model Formulation:***

To model the joint dynamics of HIV and FGS, we first developed a model for *Schistosoma haematobium* dynamics. We considered an age-structured model where the population is subdivided into two age-groups: child- and adult-group. The child-group is composed of individuals younger than 15 years old, and the adult-group is composed of individuals aged 15 years old and above. These two age-groups differ in their risk for schistosomiasis infection and their schistosomiasis prevalence. The population dynamics is modeled using a compartmental aging system of differential equations:

$$\frac{dX_{c}}{dt}=\sigma_{b}X_{a}-(\mu_{c}+\tau)X_{c} (1.1)$$

$$\frac{dX_{a}}{dt}=\tau X_{c}-\mu_{a}X_{a} (1.2)$$

where $X_{c}$ and $X_{a}$are, respectively, the total child and adult populations. The per capita birth rate is$\sigma_{b}$, $\tau$represents the transition (aging) rate, and$\mu_{c}$and $\mu_{a}$represent the child and adult mortality rates, respectively. Mortality from *S. haematobium* is negligible.^3^ We modeled schistosomiasis dynamics using the following model:

$\frac{dX_{c}^{F}}{dt}=\sigma_{b}N/2-(\mu_{c}+\tau+A_{c}(Y_{c}^{M}+Y_{c}^{F}+Y_{a}^{M}+Y_{a}^{F}))X_{c}^{F}+\gamma Y_{c}^{F}$ (2.1)

$\frac{dX_{c}^{M}}{dt}=\sigma_{b}N/2-(\mu_{c}+\tau+A_{c}(Y_{c}^{M}+Y_{c}^{F}+Y_{a}^{M}+Y_{a}^{F}))X_{c}^{M}+\gamma Y_{c}^{M}$ (2.2)

$\frac{dY_{c}^{F}}{dt}=A_{c}(Y_{c}^{M}+Y_{c}^{F}+Y_{a}^{M}+Y_{a}^{F})X_{c}^{F}-(\gamma+\tau+\mu_{c})Y_{c}^{F}$ (2.3)

$\frac{dY_{c}^{M}}{dt}=A_{c}(Y_{c}^{M}+Y_{c}^{F}+Y_{a}^{M}+Y_{a}^{F})X_{c}^{M}-(\gamma+\tau+\mu_{c})Y_{c}^{M}$ (2.4)

$\frac{dX_{a}^{F}}{dt}=\tau X_{c}^{F}-(\mu_{a}+A_{a}(Y_{c}^{M}+Y_{c}^{F}+Y_{a}^{M}+Y_{a}^{F}))X_{a}^{F}+\gamma Y_{a}^{F}$ (2.5)

$\frac{dX_{a}^{M}}{dt}=\tau X_{c}^{M}-(\mu_{a}+A_{a}(Y_{c}^{M}+Y_{c}^{F}+Y_{a}^{M}+Y_{a}^{F}))X_{a}^{M}+\gamma Y_{a}^{M}$ (2.6)

$\frac{dY_{a}^{F}}{dt}=\tau Y_{c}^{F}-(\mu_{a}+\gamma)Y_{a}^{F}+A_{a}(Y_{c}^{M}+Y_{c}^{F}+Y_{a}^{M}+Y_{a}^{F})X_{a}^{F}$ (2.7)

$\frac{dY_{a}^{M}}{dt}=\tau Y_{c}^{M}-(\mu_{a}+\gamma)Y_{a}^{M}+A_{a}(Y_{c}^{M}+Y_{c}^{F}+Y_{a}^{M}+Y_{a}^{F})X_{a}^{M}$ (2.8)

where $\gamma^{-1}$ is the duration of schistosomiasis infection, $A_{c}$ is the transmission rate to children, $A_{a}$ is the transmission rate to adults. $N$ is the total population size. As *S. haematobium* is endemic in Zimbabwe, we parameterized the schistosomiasis transmission rate by running the model to equilibrium and using least-squares approach to fit the equilibrium prevalence of *S. haematobium* predicted by the model to epidemiological studies of rural Zimbabwean (58% for school-age children and 25% for adults).^4-7^

We integrated our calibrated *S. haematobium* transmission model with HIV transmission to generate a co-infection model the HIV–FGS dynamics with the adult population. We assumed that women enter the model either infected with FGS, or uninfected. From ages 15 to 49, women can acquire FGS and/or HIV with different forces of infection. As FGS is a persistent manifestation of *S. haematobium* in endemic areas,^8,9^ we assume that there is no natural recovery from FGS among adult women, as supported from clinical data.^10^ Following Hallett et al,^11^ we developed a model for heterosexual spread of HIV, stratifying both gender and sexual activity.

The model is defined by a set of ordinary differential equations. The state variables are given by $X_{k,l}^{S,P}$: $k$is gender (1 = female, 2 = male), $l$is the sexual activity group (1 = high risk, 2 = low risk) defined according to rate of sexual partner change, $S$is the HIV infection status (1 = susceptible, 2 = infected), and $P$is the FGS infection status (1 = non-infected, 2 = infected). For men $P$is always equal to 1.

$\frac{dX_{k,l}^{1,1}}{dt}=(1-P_{G})\varphi_{k,l}(\frac{\mu+\alpha}{2})X_{\bullet,\bullet}^{\bullet,\bullet}-\lambda_{k,l}^{1}X_{k,l}^{1,1}-(\mu+\zeta_{g})X_{k,l}^{1,1}$ (3.1)

$\frac{dX_{k,l}^{1,2}}{dt}=P_{G}\varphi_{k,l}(\frac{\mu+\alpha}{2})X_{\bullet,\bullet}^{\bullet,\bullet}-\lambda_{k,l}^{2}X_{k,l}^{1,2}-\mu X_{k,l}^{1,2}+\zeta_{g}X_{k,l}^{1,1}$ (3.2)

$\frac{dX_{k,l}^{2,1}}{dt}=\lambda_{k,l}^{1}X_{k,l}^{1,1}-(\mu+\sigma+\zeta_{g})X_{k,l}^{2,1}$ (3.3)

$\frac{dX_{k,l}^{2,2}}{dt}=\lambda_{k,l}^{2}X_{k,l}^{1,2}-(\mu+\sigma)X_{k,l}^{2,2}+\zeta_{g}X_{k,l}^{2,1}.$ (3.4)

We demote by$P_{G}$ the FGS prevalence among girls age 15.$P_{G}=C_{S}\kappa_{C}$, where $C_{S}$denotes the prevalence of *S. haematobium* among girls age15 and $\kappa_{C}$is the proportion of those for whom FGS has manifested by age 15. We assumed adult women, infected with *S. haematobium*, who did not develop FGS during their childhood may acquire FGS in adulthood at a rate$\zeta_{g}$. $\zeta_{g}=A_{S}\kappa_{A}$ where $A_{S}$denotes the prevalence of *S. haematobium* among adult women and $\kappa_{A}$is the rate at which women without FGS would acquire FGS as a result of *S. haematobium* infection. *S. haematobium* prevalences were derived from the *S. haematobium* dynamic model: Eqs (2.1)–(2.8). The mean duration of sexual activity is given by $\mu^{-1}$, $\varphi_{k,l}$is the fraction of men and women, respectively, becoming sexually active in each sexual activity group. The force of infection for individuals in each gender and sexual activity group is given by $\lambda_{k,l}^{P}$.

The force of infection is calculated on the basis of the rate of partnership change, HIV prevalence among their sexual partners, and the number of sex acts in each partnership.

$\lambda_{k,l}^{P}=C_{k,l}\sum_{l'=1}^{2} (\rho_{k,l,l'}(1-(1-\beta_{k'}^{P})^{n_{L}})\frac{X_{k',l'}^{2,\bullet}}{X_{k',l'}^{\bullet,\bullet}})$ (4.1)

$L=\max(l,l')$, where $L$denotes whether a partnership is high-risklow-risk (the prime denotes that the index relates to those of the opposite gender). The number of sex acts in a partnership is denoted by $n_{L}$. The HIV transmission rate per sexual act for individuals of gender $k$and FGS infection status $P$is denoted $\beta_{k}^{P}$. We set HIV transmission rate from male-to-female to be equal to twice the transmission rate from female-to-male, $\beta_{1}^{1}=2\beta_{2}^{1}$ , in the absence of FGS.^12,26^ In the presence of FGS, we set$\beta_{1}^{2}=2c_{G}\beta_{2}^{2}$, where $c_{G}$ is the enhancement of HIV transmission rate per sexual act due to FGS. We also assume the HIV transmission rate per sexual act do not vary with risk activity group, $\beta_{2}^{1}=\beta_{2}^{2}$. We denote by $\rho_{k,l,l'}$the fraction of partnerships that individuals in the $k^{th}$gender and $l^{th}$sexual activity group form with those of the opposite gender in the $l'^{th}$activity group.

$\rho_{k,l,l'}=\varepsilon\delta_{l,l'}+(1-\varepsilon)\frac{W_{k',l'}}{\sum_{l'} W_{k',l'}}$ (5.1)

Here, $\delta_{l,l'}$is the Kronecker delta: $\delta_{i,j}=1, if i=j$ and$\delta_{i,j}=0, if i\neq j$. We assumed that men and women form partnerships such that a fraction, $\varepsilon$, of their partnership are with members of the same activity group. The remainder is randomly distributed among those of opposite gender, according to the number of partnerships available. We denote by $W_{k,l}$the total number of sexual partnerships dependent on gender $k$and sexual activity group $l$: $W_{k,l}=C_{k,l}\sum_{S} \sum_{P} X_{k,l}^{S,P}$. We denote by $C_{k,l}$the rate at which individual of gender $k$and sexual activity group $l$form partnerships.

(6.1)

$\bar{C}_{k}$is the average rate of partner change for gender $k$, and $\varpi_{k,l}$is the relative partner change for those in high risk group ($\varpi_{k,1}$) to those in the low risk group ($\varpi_{k,2}=1$).

In the case that there is a discrepancy between men in risk group $l$ and women in group $l'$regarding the number of partnerships to be formed between the risk groups, i.e. $\rho_{2,l,l'}\neq\rho_{1,l',l}$, the discrepancy is balanced according to the parameter $\psi$as:

$$D_{a,b}=\frac{W_{2,l}\rho_{2,l,l'}}{W_{1,l}\rho_{1,l',l}}$$

$$\rho_{2,l,l'}\to\rho_{2,l,l'}(D_{a,b})^{\psi-1}$$

$$\rho_{1,l',l}\to\rho_{1,l',l}(D_{a,b})^{\psi}$$

$\psi$is the extent to which the pattern of partnership formation is governed by the parameters estimated from men’s reported sexual behavior (see Garnett and Anderson^13^ for details).

To allow for behavioral response to the HIV epidemic, we assumed that the mean partner change rate varies with the number of HIV deaths per unit time: , where$\bar{c}_{k}^{*}$ is the initial contact rate,$\eta$ is the rate at which partner change declines, $D$ is the number of HIV/AIDS related deaths per year, and$N$is the total population size.

***Model Fitting:***

In a cross-sectional study, Kjetland et al identified the prevalences of HIV and FGS, as well as the odds ratio of having HIV with or without FGS (Table A1).^1^ We developed a likelihood function by assuming normal distributions for HIV and FGS prevalence and lognormal distribution for the odds ratio (Table A1). The choice of the distribution was informed by empirical data from the cross-section study.^1^ Given that no prior information is available on the level of increase risk of HIV infection per sexual act for FGS infected women ($c_{G}$), we used an objective prior (uniform distribution) and a realistic range of values (0–20) to describe the prior distribution of$c_{G}$. For all other parameters of the HIV-FGS model, prior distributions were determined from the literature (Table A2).

To mimic the Zimbabwean clinical studies, for each iteration of parameters, we ran the models for 20 years from the initial conditions, simulating the start of the HIV epidemic in approximately 1980 until 1999 when Kjetland et al studies were completed.^1,2^ We ran five separately initialized Markov Chain Monte Carlo (MCMC) simulations for 300,000 iterations each using the Metropolis-Hastings method. Convergence was assessed using the Brooks-Gelman-Rubin diagnostic criterion.^14^

***Interventions:***

We modeled two potential scenarios for the effect of mass praziquantel administration on reducing HIV transmission. We assumed that treatment is annually administered to school-age children (5 to 14 years old). In the first scenario, we assumed that women who have received treatment during their childhood have a reduced FGS prevalence relative to those who did not receive treatment. We assumed treatment would reduce FGS prevalence among 15 years old girls by $ep$(30%–70%), in the treated population.^8^ In the second scenario, we assumed that for women who received treatment during their childhood, treatment will mitigate their risk of HIV transmission by $er$(30%–70%). The second scenario extends the first by considering a situation in which mass treatment does not necessarily reduce FGS prevalence among treated women, but rather reduces the manifestations of FGS regarded as exacerbating risk of HIV acquisition.

From the time that intervention is initiated, the model is run for the duration of the intervention either with or without mass praziquantel administration. The cost of each intervention is found by multiplying the discounted price of treatment by the number of treated individuals. The costs were discounted at a 3% annual rate as recommended by the Panel on Cost-Effectiveness in Health and Medicine of the US Public Health Service and the WHO.^15^

***Sensitivity Analysis***

To identify the contribution of each of the estimated input parameters to the variability of the outcome measures (number of HIV cases averted, and averted medical care costs) of our model, we calculated the partial rank correlation coefficients (PRCCs).^16^ PRCC quantifies the degree of monotonicity between a specific input parameter and an outcome measure. In order to conduct the sensitivity analysis, we used a Latin Hypercube procedure to draw 10,000 sample values for the input parameters of our model.^17^

**Table A1**: Kjetland data – Confidence Interval (CI), and Distribution approximation

| **Statistic** | **Values** | **Modeled Distribution** |
| --- | --- | --- |
| Prevalence of HIV | 28% (CI: 24% - 32%) | Mean 0.28, SE 0.021 (Normal) |
| Prevalence of FGS | 46% (CI: 42% - 50%) | Mean 0.46, SE 0.021 (Normal) |
| Odds Ratio | 2.1 (CI: 1.2 – 3.5) | Mean 2.1,SE 0.352 (Lognormal) |

**Table A2: Parameter definitions of Scistosomiasis Dynamic Model**

|  |  |  | | |  |
| --- | --- | --- | --- | --- | --- |
| **Parameter** | **Definition** | **Value** | **Ref** | | |
| $\sigma_{b}$ | Per capita birth rate | 0.034 yr^-1^ | | [27] | |
| $\mu_{c}$ | Child mortality rate | 0.02 yr^-1^ | | [18] | |
| $\mu_{a}$ | Adult mortality rate | 0.02 yr^-1^ | | [18] | |
| $\tau$ | Aging rate from youth to adulthood | 1/10 yr^-1^ | | --- | |
| $A_{c}$ | Children transmission rate | 0.38 | | estimated* | |
| $A_{a}$ | Adult transmission rate | 0.04 | | estimated* | |

*Parameters were estimated suing least-squares to fit the *S. haematobium* dynamic model to Zimbabwean prevalence data.

b

b

### References:

1. Kjetland EF, Ndhlovu PD, Gomo E, et al. Association between genital schistosomiasis and HIV in rural Zimbabwean women. AIDS, 2006; **20**: 593-600.

2. Kjetland EF, Ndhlovu PD, Mduluza T, et al. Simple clinical manifestations of genital schistosoma haematobium infection in rural zimbabwean women. Am J Trop Med Hyg 2005; **72**(3): 311–19.

3. Van der werf M, de Vlas SJ, Brooker S, et al. Quantification of clinical morbidity associated with schistosome infection in sub-Saharan Africa. Acta Trop 2003; **86**: p. 125-39.

4. Midzi N, Mtapuri-Zinyowera S, Mapingure MP, Paul NH, Sangweme D, Hlerema G, et al. Knowledge Attitudes and Practices of grade three primary schoolchildren in relation to schistosomiasis, soil transmitted helminthiasis and malaria in Zimbabwe. BMC Infect Dis 2011; **11**(1): 169.

5. Midzi N, Sangweme D, Zinyowera S, Mapingure MP, Brouwer KC, Munatsi A, Mutapi F, Mudzori J, Kumar N, Woelk G, et al. The burden of polyparasitism among primary schoolchildren in rural and farming areas in Zimbabwe. Trans R Soc Trop Med Hyg. 2008; **102**(10):1039-45

6. Taylor P, Makura O. Prevalence and distribution of schistosomiasis in Zimbabwe. Ann Trop Med Parasitol 1985; **79**(3): 287–99.

7. Ndhlovu PD, Mduluza T, Kjetland EF, Midzi N, Nyanga L, et al. Prevalence of urinary schistosomiasis and HIV in females living in a rural community of Zimbabwe: does age matter? Trans Roy Soc Trop Med Hyg 2007; **101**: 433–8.

8. Kjetland EF, Ndhlovu PD, Kurewa EN, et al., Prevention of gynecologic contact bleeding and genital sandy patches by childhood anti-schistosomal treatment. Am J Trop Med Hyg 2008; **79**(1): 79–83.

9. Satayathum SA, Muchiri EM, Ouma JH, Whalen CC, King CH. Factors affecting infection or reinfection with Schistosoma haematobium in coastal Kenya: survival analysis during a nine-year, school-based treatment program. Am J Trop Med Hyg. 2006; **75**(1):83-92.

10. Poggensee G, Kiwelu I, Weger V , et al. Female genital schistosomiasis of the lower genital tract: prevalence and disease-associated morbidity in northern Tanzania. J Infect Dis, 2000; **181**(3):1210-3.

11. Hallett TB, Gregson S, Mugurungi O, Gonese E, and Garnett GP. Assessing evidence for behaviour change affecting the course of HIV epidemics: A new mathematical modelling approach and application to data from Zimbabwe. Epidemics 2009; **1**: p. 108-117.

12. Bioly M-C, Baggaley R, Wang L, Masse B, While RG, Hayes RJ, et al. Heterosexual risk of HIV-1 infection per sexual act: systematic review and meta-analysis of observational studies. Lancet Infect Dis 2009; **9**: 118–129

13 Garnett GP, Anderson RM: **Balancing sexual partnerships in an age and activity stratified model of HIV transmission in heterosexual populations.** IMA J Math Appl Med Biol 1994, **11**:161-192.

14 Brooks, S. and Gelman, A. General methods for monitoring convergence of iterative simulations. J Comput Graph Stat 1998; **7**:434-455.

15. Gold M, Siegel JE, Russell LB, and Weinstein MC. Cost-effectiveness in health and medicine. New York: Oxford University Press. 1996; 425.

16. Blower SM, Dowlatabadi H. Sensitivity and uncertainty analysis of complex-models of disease transmission - an HIV model, as an example. Int Stat Rev 1994; 6(2):229–43.

17. Iman RL, Helton JC, [Campbell JE.](http://en.wikipedia.org/wiki/James_Edward_Campbell) An approach to sensitivity analysis of computer models, Part 1. Introduction, input variable selection and preliminary variable assessment. Journal of Quality Technology 1981; **13** (3): 174–183.

**18**. United Nations. (2008) World urbanization prospects: the 2007 revision. New York: United Nations. (Accessed June 3, 2011, at <http://www.un.org/esa/population/publications/wup2007/2007WUP_ExecSum_web.pdf>)

19 King CH, Olbrych SK, Soon M, Singer ME, Carter J, Colley DG. Utility of repeated praziquantel dosing in the treatment of schistosomiasis in high-risk communities in Africa: a systematic review. PLoS Negl Trop Dis. 2011;**5**(9):e1321

20. Goldman AS, Guisinger VH, Aikins M, Amarillo ML, Belizario VY, et al. National mass drug administration costs for lymphatic filariasis elimination. PLoS Negl Trop Dis 2007; **1**: e67

21. Guyatt H. (2003) The cost of delivering and sustaining a control programme for schistosomiasis and soil-transmitted helminthiasis. Acta Trop; 86: 267–74.

22. World Health Organization. Towards universal access: scaling up priority HIV/AIDS interventions in the health sector: progress report 2010. Geneva: World Health Organization; 2010. (Accessed June 3, 2011, at http://www.who.int/hiv/pub/2010progressreport/en/)

23. Amico P, Aran C, Avila C. HIV spending as a share of the total health expenditure: An analysis of regional variation in a multi-country study. PLoS One 2010; **5**(9):e12997.

24. World Health Organization. Global health expenditure database (accessed November 8, 2011 at www.who.int/nha/database/).

25. Stover J, Bertozzi S, Gutierrez JP, Walker N, Stanecki KA, Greener R, et al. The global impact of scaling up HIV/AIDS prevention programs in low- and middle-income countries. Science 2006; **311**:1474–6.

26. Hughes JP, Baeten JM, Lingappa JR, Magaret AS, Wald A, de Bruyn G, et al. Determinants of per-coital-act HIV-1 infectivity among African HIV-1-serodiscordant couples. JID 2012; **205**: 358–365

27. United Nations. (2008) World urbanization prospects: the 2007 revision. New York: United Nations. (Accessed June 3, 2011, at http://www.un.org/esa/population/publications/wup2007/2007WUP_ExecSum_web.pdf)

# Supplementary table

1. **Table S1:** **Base line estimate and distributions of selected model variables**

| **Variable** | **Base value** | **Distribution** | **Ref** |
| --- | --- | --- | --- |
| Cost per praziquantel tablet (600 mg tablet) | US$0.08 | NA | [19] |
| Cost of delivery of praziquantel per individuals | US$0.21 | Min 0.06, Max 2.23 (Uniform) | [20,21] |
| ART coverage (proportion HIV patients receiving antiretroviral therapy)^¥^ | 0.34 | Mean 0.34, SD 0.02 (Normal) | [22] |
| Zimbabwe non-HIV/AIDS health expenditure (cost per person per annum) | US$26 | Mean 26, SD 4.8 (Gamma) | [23,24] |
| Cost lifetime ART (ARV first line, second line, ARV monitoring) | US$3000 | NA | [25] |
| Other lifetime cost of HIV treatment (prophylaxis and treatment of opportunistic infections, diagnostic and routine testing, palliative care) | US$695 | NA | [25] |

SD: standard deviation; ¥ ART = Antiretroviral Therapy
